# Some Prolegomena to a Philosophy of Medicine

**Published:** 1896-06-27

**Authors:** 


### Editor's Letter-Box.

[Our correspondents are reminded that prolixity is a great bar to publication, and that brevity of style and conciseness of statement greatly facilitate early insertion.]

### SOME PROLEGOMENA TO A PHILOSOPHY OF MEDICINE.

We have received from Dr. Charles W. Hayward, of Liverpool, a strong protest—perhaps we may even say a violent and abusive one—against the tone taken in regard to homœopathy, in a review of a book by Dr. Goldsbrough, entitled "Some Prolegomena to a Philosophy of Medicine," which appeared in The Hospital for June 6th. We can hardly suppose that Dr. Hayward really expected us to publish his letter, nor do we feel called upon to do so, for although he protests strongly against the strictures made in regard to homœopathy, he has not much to say in defence of the book itself. Our reviewer said, "But what has a man to do with 'philosophising' if he has not attained the elementary art of setting forth his meaning with clearness and precision?" Dr. Hayward says: "Dr. Goldsbrough's reasoning is, I agree, difficult to follow, and one is inclined to hope that the philosophy of medicine, to which his remarks are 'prolegomena,' may be more understandable. If it is not, I grieve for the future of philosophical medicine." There does not seem much to choose between the two.

